# A novel risk factor for dementia: chronic microplastic exposure

**DOI:** 10.3389/fneur.2025.1581109

**Published:** 2025-05-30

**Authors:** Elif Gecegelen, Mete Ucdal, Burcu Balam Dogu

**Affiliations:** ^1^Division of Geriatric Medicine, Department of Internal Medicine, Hacettepe University, Ankara, Türkiye; ^2^Department of Internal Medicine, Hacettepe University, Ankara, Türkiye

**Keywords:** dementia, microplastics, neuroinflammation, oxidative stress, blood–brain barrier, amyloid-beta aggregation, environmental pollutants

## Abstract

Recent advances in dementia research have expanded our understanding of modifiable risk factors, with air pollution being a well-established contributor. However, microplastics—plastic particles smaller than 5 mm—represent an understudied component of environmental pollution that may significantly impact neurological health. This review examines emerging evidence linking chronic microplastic exposure to increased dementia risk. Microplastics enter the human body through multiple routes, including ingestion of contaminated food and water, inhalation, and dermal absorption, with demonstrated ability to cross the blood–brain barrier and initiate several pathogenic pathways. Four primary mechanisms appear to mediate microplastic-induced neurodegeneration: increased oxidative stress through reactive oxygen species (ROS) production; neuroinflammation via microglial activation and chronic inflammatory responses; neurotoxicity from transported persistent organic pollutants (POPs) and heavy metals; and accelerated amyloid-beta pathology through enhanced Aβ40 and Aβ42 nucleation. Recent bioaccumulation studies have revealed significantly elevated microplastic concentrations in the brains of dementia patients compared to non-dementia controls, supporting a potential dose-dependent relationship. Sources of environmental microplastics include industrial waste, synthetic textiles, plastic degradation products, and tire wear particles, creating a ubiquitous exposure risk through contaminated air, food, and water. While preliminary evidence supports a mechanistic link between microplastics and neurodegeneration, comprehensive epidemiological studies with larger datasets are needed to quantify this relationship and establish dose–response patterns. Future research should focus on identifying which microplastic types pose the greatest neurological risks, determining threshold exposure levels, and developing interventions to mitigate exposure.

## 1 Introduction

Dementia represents a significant public health challenge in the 21st century, currently affecting approximately 55 million individuals worldwide, with projections indicating this number may nearly triple by 2050. Recent advancements in dementia research have considerably enhanced our understanding of various modifiable risk factors associated with this condition. Current evidence identifies multiple potentially modifiable risk factors for dementia development, including sensory impairments (hearing loss), cardiovascular factors (hypertension), lifestyle behaviors (smoking, obesity, physical inactivity), metabolic conditions (diabetes), psychological factors (depression), social determinants (isolation), and environmental exposures (air pollution). These findings highlight the multifactorial etiology of dementia and underscore the importance of comprehensive preventive strategies addressing these diverse risk domains (1). This evolving understanding underscores the multifactorial nature of dementia pathogenesis and highlights the importance of environmental factors in neurodegenerative processes.

Among environmental risk factors, air pollution has emerged as a significant contributor to dementia risk. Numerous epidemiological studies have demonstrated associations between atmospheric particulate matter (PM₂.₅ and PM₁₀) exposure and increased incidence of dementia, Alzheimer’s disease, and mild cognitive impairment. However, the specific components of air pollution responsible for neurotoxic effects remain incompletely characterized (2).

Microplastics represent an increasingly ubiquitous environmental contaminant that has received limited attention in dementia research. Defined as plastic particles smaller than 5 mm, microplastics have infiltrated virtually every environmental compartment, including air, water, and soil (3). Recent evidence demonstrates that microplastics can enter the human body through multiple routes, cross biological barriers including the blood–brain barrier, and potentially initiate or exacerbate pathological processes linked to neurodegenerative diseases (4).

This review examines the emerging evidence linking chronic microplastic exposure to increased dementia risk, building upon established connections between air pollution and cognitive decline. We explore the sources and routes of microplastic exposure, mechanisms of microplastic-induced neurotoxicity, and recent findings regarding microplastic bioaccumulation in human brain tissue. Additionally, we identify critical knowledge gaps and future research directions necessary to establish microplastics as a potential modifiable risk factor for dementia.

## 2 Sources and routes of microplastic exposure

### 2.1 Environmental sources of microplastics

Microplastics represent a heterogeneous class of synthetic particulate matter, taxonomically categorized into two distinct classifications based on their origin and production pathway. Primary microplastics are intentionally manufactured at microscopic dimensions (<5 mm) for specific commercial and industrial applications, including microbeads in personal care formulations, pre-production plastic pellets (nurdles), and engineered microspheres for various technological applications (5). Secondary microplastics, conversely, originate through the progressive fragmentation and degradation of larger plastic items following their introduction into environmental matrices. These degradation processes involve complex physicochemical mechanisms including photo-oxidation, mechanical abrasion, hydrolytic cleavage, and microbial biodegradation, resulting in increasingly diminished particle dimensions while maintaining polymer integrity (6).

Industrial manufacturing and processing facilities constitute significant point sources of environmental microplastic contamination through multiple emission pathways. These facilities release microplastic particles via wastewater discharge streams, atmospheric emissions from thermal processes, and fugitive particle release during material handling operations (7). Polymer production facilities, plastics fabrication operations, and various industrial processes involving synthetic polymeric materials contribute substantially to environmental microplastic burden through both intentional and unintentional release mechanisms (8). Quantitative assessments of industrial contributions to environmental microplastic contamination reveal disproportionate contributions from polymer processing facilities, with emission rates varying significantly according to operational parameters, pollution control technologies, and regulatory compliance frameworks. The physicochemical characteristics of these industrially-derived microplastics frequently reflect their manufacturing origins, exhibiting uniformity in composition, morphology, and additive profiles (9).

Synthetic textile materials represent a pervasive and persistent source of microplastic fibers throughout the global ecosystem. Garments and textiles composed of synthetic polymers including polyester, nylon, acrylic, and polyamide release microfibers during various phases of their lifecycle, with particular emission intensity during manufacturing processes, consumer laundering operations, and physical abrasion during wear (10). Quantitative investigations have demonstrated that a single domestic washing cycle of synthetic clothing items can release between 1,900 and 700,000 microfibers into wastewater systems, with variation dependent upon fabric composition, textile construction parameters, washing conditions, and garment age (11). These microfibers exhibit distinctive morphological characteristics, typically presenting as elongated cylindrical structures with high aspect ratios, which influences their environmental transport dynamics, bioavailability, and ecological interactions. The global proliferation of synthetic textiles in consumer products has established this source category as a ubiquitous contributor to environmental microplastic contamination in both terrestrial and aquatic ecosystems (12).

Environmental degradation of macroplastic debris constitutes a substantial ongoing source of secondary microplastics across diverse ecological compartments. Larger plastic items introduced into the environment undergo progressive weathering and fragmentation through exposure to ultraviolet radiation, thermal fluctuations, mechanical forces, and microbial colonization, generating an expanding inventory of secondary microplastic particles. Common source materials include improperly disposed consumer packaging, single-use plastic items, abandoned fishing gear, agricultural films, and deteriorating plastic infrastructure components. The fragmentation kinetics and resultant particle characteristics demonstrate high variability depending on polymer composition, environmental conditions, and exposure duration (13). These degradation-derived microplastics frequently exhibit irregular morphologies, weathered surfaces, and altered polymer properties resulting from oxidative processes and leaching of additives. The environmental persistence of many common polymers ensures that this fragmentation process represents a continuing source of microplastic generation for decades to centuries following initial environmental introduction (14).

Tire wear particles (TWPs) constitute a significant but frequently overlooked source of microplastic contamination in urban and roadside environments. Vehicle tire abrasion during normal operational use releases complex microplastic particles composed not only of styrene-butadiene rubber polymers but also incorporating carbon black, silica reinforcing agents, processing oils, vulcanization accelerators, antioxidants, and various performance-enhancing additives. These particles exhibit distinctive physicochemical properties, including irregular morphology and high sorptive capacity for co-pollutants. Quantitative analyses indicate that tire wear contributes substantially to roadside dust composition and urban particulate air pollution, with emission factors ranging from 0.1 to 0.4 g/vehicle-kilometer depending on driving conditions, road surface characteristics, and tire composition (15). The environmental fate of these particles involves atmospheric transport, surface runoff into aquatic systems, and accumulation in roadside soils, presenting multiple exposure pathways for biotic receptors including humans. The complex chemical composition of TWPs and their potential for co-transport of adsorbed pollutants presents distinctive toxicological concerns compared to other microplastic sources (16).

**Figure 1:**
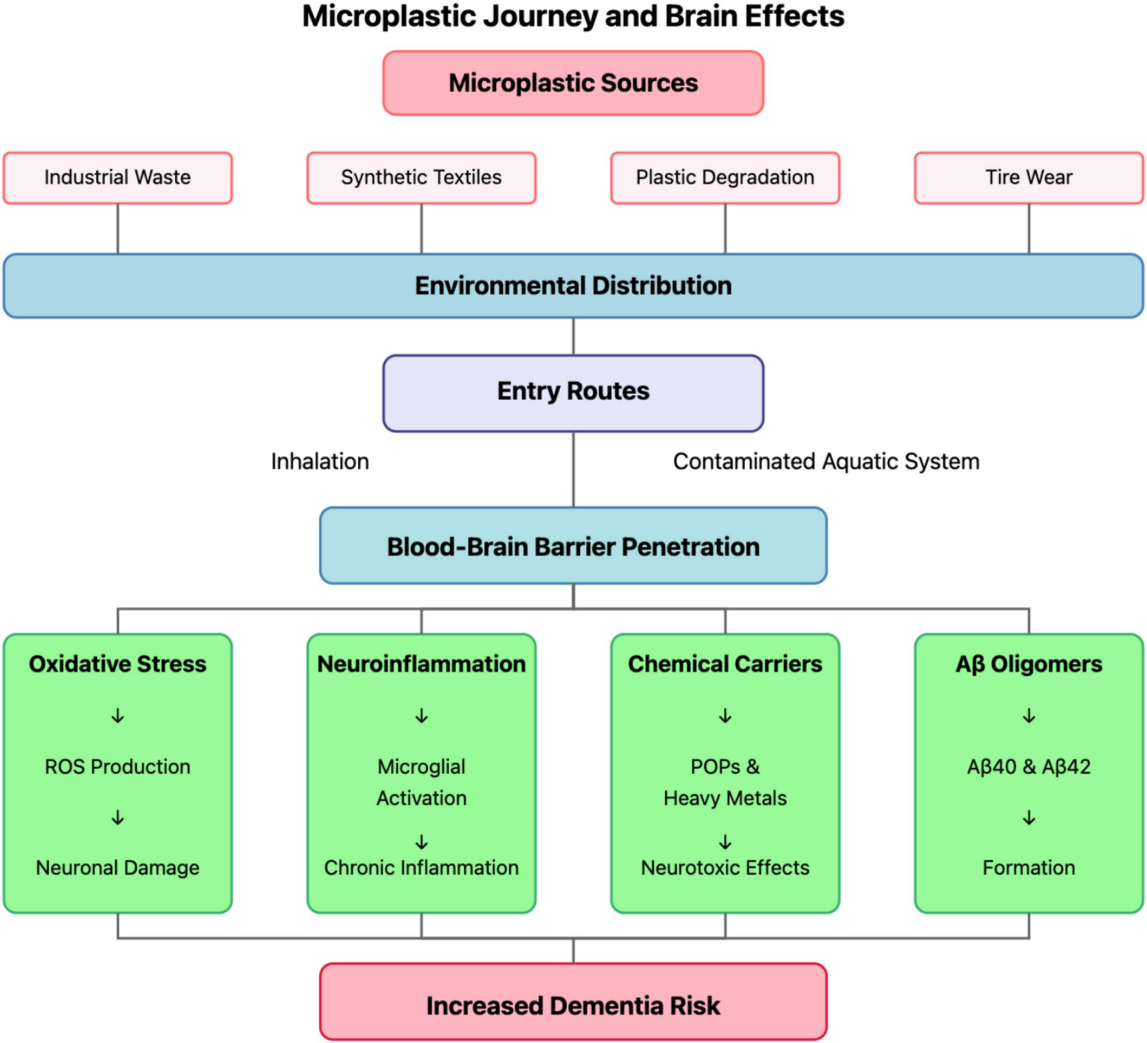
Microplastic journey and brain effects. The diagram illustrates the comprehensive pathway of microplastics from their primary sources (industrial waste, synthetic textiles, plastic degradation, and tire wear) through environmental distribution to human exposure routes (inhalation and contaminated aquatic systems). Following blood–brain barrier penetration, microplastics trigger four major pathogenic mechanisms (oxidative stress, neuroinflammation, chemical carriers, and Aβ oligomer formation), ultimately contributing to increased dementia risk through neuronal damage, chronic inflammation, neurotoxic effects, and Aβ40/42 formation.

Personal care products historically incorporated intentionally manufactured primary microplastics as functional ingredients, particularly in exfoliating cleansers, toothpastes, and cosmetic formulations. These engineered microbeads, predominantly composed of polyethylene, polypropylene, and polyethylene terephthalate, served as mechanical exfoliants, opacifying agents, film-formers, and viscosity modifiers in consumer product formulations. A single cosmetic product could contain between 5,000 and 95,000 microplastic particles, presenting a direct pathway for environmental introduction following consumer use and wastewater discharge (17). Although regulatory restrictions in numerous jurisdictions have progressively limited or prohibited these applications, the historical introduction of these materials and their continued use in regions lacking regulatory oversight maintains their relevance as a microplastic source. Unlike environmentally degraded secondary microplastics, these primary microbeads exhibit uniform spherical morphologies, consistent size distributions, and homogeneous composition, characteristics that influence their environmental transport behavior and biological interactions (18). Figure 1 illustrates the comprehensive journey of microplastics from their environmental sources to their pathogenic effects in the brain, highlighting the multiple exposure pathways and biological mechanisms discussed in subsequent sections.

## 3 Mechanisms of microplastic-induced neurodegeneration

### 3.1 Oxidative stress pathway

Oxidative stress represents a fundamental pathophysiological mechanism through which microplastic exposure may exert neurodegenerative effects. This process occurs when cellular production of reactive oxygen species (ROS) exceeds the intrinsic antioxidant defense capacity, resulting in oxidative damage to cellular macromolecules including membrane lipids, structural and functional proteins, and nucleic acids. Contemporary experimental investigations have consistently demonstrated that microplastic particles induce significant ROS generation across diverse cell lineages, with neuronal populations exhibiting particular susceptibility to oxidative perturbations (19). The temporal dynamics of ROS production following microplastic exposure display both acute elevations and sustained oxidative disturbances, suggesting mechanisms for both immediate cytotoxicity and chronic neurodegeneration (20).

The molecular mechanisms underlying microplastic-induced oxidative stress involve multiple intersecting pathways with synergistic effects on cellular redox homeostasis. Surface physicochemical properties of microplastic particles, particularly those exhibiting reactive functional groups resulting from environmental weathering and degradation processes, demonstrate direct catalytic capacity for ROS formation through electron transfer reactions and interaction with transition metal ions present in biological systems. These surface-mediated reactions generate superoxide anions, hydrogen peroxide, and hydroxyl radicals, which subsequently propagate oxidative damage through chain reactions with cellular components. Notably, the reactive surface area-to-volume ratio increases exponentially as microplastic particles fragment into nanoscale dimensions, potentially amplifying their oxidative potential when degraded to sizes compatible with cellular internalization (21).

Microplastic exposure additionally induces profound mitochondrial dysfunction, disrupting the primary cellular energy-generating organelle and a critical regulator of redox homeostasis. Experimental evidence indicates that internalized microplastic particles impair electron transport chain efficiency, disrupt mitochondrial membrane potential, and compromise ATP synthesis capacity. These mitochondrial perturbations result in electron leakage from respiratory complexes, generating excessive superoxide radicals while simultaneously compromising energy availability. This bioenergetic-oxidative coupling creates a particularly deleterious environment for neuronal populations, which maintain exceptionally high metabolic demands and limited glycolytic capacity, rendering them vulnerable to mitochondrial impairment and subsequent oxidative damage (22).

Further exacerbating cellular oxidative stress, microplastic exposure significantly depletes endogenous antioxidant defense systems. Experimental investigations have documented reduced activity and expression of critical antioxidant enzymes including superoxide dismutase (SOD), catalase, glutathione peroxidase, and glutathione reductase following microplastic challenge. Concomitantly, cellular concentrations of non-enzymatic antioxidants such as reduced glutathione (GSH) demonstrate marked depletion, compromising the redox buffering capacity of affected cells. This impairment of antioxidant systems occurs through multiple mechanisms, including direct inhibition of enzymatic activity, transcriptional downregulation, and consumption of antioxidant reservoirs in response to excessive ROS generation (19, 23).

The central nervous system exhibits heightened vulnerability to oxidative perturbations due to several intrinsic characteristics: exceptionally high oxygen consumption rates (consuming approximately 20% of total body oxygen despite representing only 2% of body mass); abundant polyunsaturated fatty acids in neuronal membranes providing readily oxidizable substrates; relatively limited antioxidant enzyme expression compared to other tissues; high transition metal content capable of catalyzing oxidative reactions; and limited regenerative capacity (19). These factors collectively establish the brain as particularly susceptible to microplastic-induced oxidative damage. Substantial evidence implicates chronic oxidative stress as a central pathogenic mechanism in multiple neurodegenerative conditions, including Alzheimer’s disease, Parkinson’s disease, and vascular dementia, suggesting a plausible mechanistic link between microplastic exposure, oxidative stress induction, and subsequent neurodegeneration (24).

Recent experimental investigations have established direct causative relationships between microplastic-induced oxidative stress and cognitive impairment, strengthening the mechanistic link to dementia pathogenesis. Kunming mice orally exposed to polystyrene microplastics (5.0–5.9 μm, 0.01–1 mg/d for 4 weeks) exhibited significantly increased hippocampal oxidative stress markers (elevated ROS, MDA; decreased GSH) concurrent with spatial memory deficits in Morris water maze assessments. Their data revealed dose-dependent increases in escape latency and progressive disorganization of hippocampal cytoarchitecture, including reduction of Nissl bodies and pyramidal neuron disruption that directly corresponded to cognitive performance decrements. Most significantly, intervention with the antioxidant Vitamin E effectively reversed both the biochemical oxidative parameters and learning deficits, establishing a mechanistic continuum between microplastic exposure, oxidative damage, and cognitive dysfunction. The study further documented microplastic-induced decreases in acetylcholine levels and CREB/BDNF pathway inhibition, demonstrating interconnected neurochemical perturbations consistent with established neurodegenerative processes (25). In human neuroblastoma cells, microplastics have been shown to cause mitochondrial dysfunction, oxidative stress, and autophagy activation, leading to apoptosis with neurotoxic effects (26). In line with these findings, Vojnits et al. (20) demonstrated that microplastics (MPs) accumulate in the mouse brain following intranasal administration and induce dose-dependent cytotoxicity in human neurons. Their study documented that smaller particles (20–100 nm) exhibit greater toxicity than larger microplastics (2 μm). Central to their mechanistic explanation was the demonstration of MP-induced mitochondrial ROS production, with the resulting neurodegeneration significantly attenuated by *N*-acetyl-l-cysteine treatment (20). The vulnerability of neurovascular units to microplastic-induced oxidative damage has been elucidated through blood–brain barrier (BBB) models, with Wang et al. demonstrating that nanoscale polystyrene particles (50 nm) compromise BBB integrity through ROS-mediated tight junction protein degradation, thereby facilitating accelerated cerebral accumulation of circulating neurotoxicants (27).

Microplastic-induced inflammation and oxidative stress can cause neuroinflammation and neurodegeneration in the brain, alter the behavioral patterns of mice, and reduce acetylcholinesterase (AChE) activity. Inhibition of AChE activity was observed when mice were allowed to inhale aerosols containing microplastics for 7 days (28). Moreover, Jin et al. (29) evaluated neurotoxicity in BALB/c mice chronically exposed to polystyrene microplastics (2–2.5 μm, 0.05 mg/g of diet for 12 weeks), reporting impaired spatial learning in the Barnes maze alongside hippocampal neuronal apoptosis, dendritic spine loss, and significantly elevated ROS and 4-HNE levels, thereby implicating oxidative stress–driven synaptic degeneration. Similarly, Chen et al. (30) showed that maternal ingestion of 50 nm polystyrene nanoplastics (1 mg/kg/day during gestation and lactation) triggers NOCA4-mediated ferritinophagy and ferroptosis in offspring hippocampi, evidenced by decreased GPX4 and GSH levels, widespread neuronal loss, and early cognitive deficits in novel object recognition tests. Collectively, these findings consolidate a mechanistic framework linking microplastic exposure, ROS-mediated neural damage, and synaptic dysfunction to cognitive decline, highlighting oxidative stress as a pivotal contributor to dementia pathogenesis and a critical target for future neuroprotective strategies.

### 3.2 Neuroinflammatory cascade

Neuroinflammation constitutes a critical pathophysiological mechanism underlying multiple neurodegenerative disorders, characterized by complex cellular and molecular responses within the central nervous system (31). Emerging evidence indicates that microplastic particles can initiate and perpetuate neuroinflammatory cascades through multiple complementary pathways, establishing a chronic inflammatory microenvironment conducive to neurodegeneration (32). The neuroinflammatory response to microplastics involves coordinated activity of resident immune cells, infiltrating peripheral immune populations, and altered neuron–glia communication networks, collectively mediating progressive neuronal dysfunction and loss (33).

Microglial activation represents the primary initiating event in microplastic-induced neuroinflammation. As the resident macrophage-like immune cells of the central nervous system, microglia function as first-line sentinels, rapidly responding to foreign particulates through pattern recognition receptors including Toll-like receptors (TLRs), scavenger receptors, and complement receptors. Recent experimental investigations utilizing high-resolution imaging techniques and flow cytometric analysis have demonstrated that polystyrene microplastic particles directly activate microglial populations, inducing characteristic morphological transformation from ramified surveillance states to amoeboid phenotypes associated with activation. This phenotypic transition is accompanied by significant alterations in microglial transcriptional profiles, surface marker expression patterns, phagocytic activity, and secretory functions. Notably, different microplastic compositions, sizes, and surface characteristics elicit distinct microglial activation patterns, suggesting polymer-specific neuroinflammatory potentials that may contribute to differential neurodegenerative risks (34).

Activated microglia following microplastic exposure release a complex array of pro-inflammatory cytokines that orchestrate subsequent neuroinflammatory amplification. Experimental evidence documents significant upregulation of interleukin-1β (IL-1β), tumor necrosis factor-*α* (TNF-α), interleukin-6 (IL-6), and interleukin-8 (IL-8) following microglial microplastic challenge. These pro-inflammatory mediators exert multiple detrimental effects within the neural parenchyma: disrupting synaptic transmission and plasticity; promoting further microglial and astrocytic activation through paracrine signaling; inducing blood–brain barrier permeabilization through tight junction disruption and matrix metalloproteinase upregulation; and recruiting peripheral immune cells that exacerbate inflammatory processes (35). Quantitative analyses reveal that both cytokine concentration and temporal expression patterns significantly influence subsequent neurodegenerative progression, with chronic low-level inflammation potentially exerting more detrimental effects than acute inflammatory responses due to the cumulative impact of sustained neuronal exposure to inflammatory mediators (36, 37).

Molecular investigations have identified the NLRP3 inflammasome pathway as a critical mechanistic link between microplastic exposure and neuroinflammatory progression. Contemporary research demonstrates that microplastic particles, particularly those in the nanoscale range, activate the NLRP3 (NOD-, LRR- and pyrin domain-containing protein 3) inflammasome complex in microglia through both direct interaction and lysosomal destabilization following phagocytic internalization. This activation initiates assembly of the multiprotein inflammasome complex, facilitating caspase-1 activation and subsequent proteolytic processing of pro-inflammatory cytokines IL-1β and IL-18 to their mature, biologically active forms. Additionally, sustained inflammasome activation triggers microglial pyroptosis, a highly inflammatory programmed cell death mechanism characterized by cellular swelling, plasma membrane rupture, and release of intracellular contents including damage-associated molecular patterns (DAMPs) that further propagate inflammatory responses. This pyroptotic cell death pathway represents a particularly detrimental component of microplastic-induced neuroinflammation, creating a feed-forward cycle of inflammation and cellular damage (38).

### 3.3 Chemical carrier function

Microplastic particles demonstrate significant capacity to function as vectors for neurotoxic environmental contaminants, effectively concentrating and transporting these compounds to neuronal populations that would otherwise experience limited exposure. This “Trojan horse” mechanism represents a distinctive aspect of microplastic neurotoxicity, wherein the polymeric particles serve as carriers for adsorbed xenobiotics that may subsequently be released within neural tissue following microplastic penetration of the blood–brain barrier. The chemical carrier functionality of microplastics encompasses multiple classes of environmental contaminants with established neurotoxic properties, potentially amplifying the neurological impact beyond the intrinsic toxicity of the microplastic particles themselves (39).

Persistent organic pollutants (POPs) exhibit pronounced affinity for microplastic surfaces due to hydrophobic interactions between these compounds and the predominantly hydrophobic polymer matrices. Quantitative partitioning studies demonstrate that microplastics efficiently adsorb and concentrate diverse POPs including polychlorinated biphenyls (PCBs), polycyclic aromatic hydrocarbons (PAHs), organochlorine pesticides, polybrominated diphenyl ethers (PBDEs), and perfluoroalkyl substances (PFAS) from environmental media (40). The partition coefficients between microplastics and surrounding aqueous environments for these compounds frequently exceed 10^4, indicating substantial bioconcentration potential. Following adsorption, these POPs may undergo subsequent desorption within biological tissues, particularly in lipid-rich environments such as the brain, where concentration gradients and local biochemical conditions facilitate pollutant mobilization from polymer surfaces (41). Many adsorbed POPs demonstrate well-characterized neurotoxic properties, including disruption of neurotransmitter systems, impairment of calcium homeostasis, induction of oxidative stress, alterations in neuronal cytoskeletal organization, and perturbation of neurodevelopmental processes (42).

Heavy metal accumulation represents another significant dimension of microplastic chemical carrier functionality. Experimental analyses demonstrate that microplastic particles can adsorb and concentrate various heavy metals including lead, mercury, cadmium, arsenic, and aluminum from aquatic and terrestrial environments through multiple binding mechanisms including electrostatic interactions, complexation with functional groups on weathered microplastic surfaces, and precipitation of metal oxides. The adsorption capacity varies substantially based on microplastic polymer composition, particle size, surface charge characteristics, and degree of environmental weathering, with oxidized microplastic surfaces typically demonstrating enhanced metal binding capacity through increased surface functionality (43). These metals exhibit well-established neurotoxicity through mechanisms including enzyme inhibition, disruption of calcium signaling, generation of reactive oxygen species, interference with neurotransmitter systems, and displacement of essential metals from metalloenzymes and structural proteins. Notably, many heavy metals demonstrate particular tropism for specific brain regions, with the hippocampus, cerebellum, and prefrontal cortex exhibiting heightened vulnerability, creating regional patterns of neurodegeneration that correlate with specific cognitive and neurological manifestations observed in dementia syndromes (44).

Microplastic particles themselves contain numerous chemical additives incorporated during manufacturing processes to modify polymer properties, many of which demonstrate neurotoxic potential. These additives include plasticizers (phthalates, adipates, and bisphenols), flame retardants (organophosphates and polybrominated compounds), stabilizers (nonylphenols and bisphenol A), colorants, and antimicrobial agents, which collectively may constitute up to 4% of plastic mass. Environmental weathering, mechanical stress, and biological degradation processes facilitate leaching of these additives from microplastic matrices, potentially releasing these compounds within neural tissue following microplastic translocation across the blood–brain barrier (45). Many plastic additives demonstrate established neurotoxicity, with particular concerns regarding their endocrine-disrupting properties that may impact neurosteroid metabolism and neuroendocrine signaling pathways critical for cognitive function. Of particular concern, several common plastic additives including bisphenol A and specific phthalate esters demonstrate blood–brain barrier permeability and capacity to modulate neurotransmitter systems including dopaminergic, glutamatergic, and cholinergic pathways implicated in cognitive function and neurodegenerative progression (46, 47).

The microplastic carrier effect may substantially amplify neurotoxic potential beyond the intrinsic toxicity of the particles themselves, creating a synergistic interaction between the physical presence of microplastics and the chemical burden they transport. When microplastic particles carrying adsorbed contaminants penetrate the blood–brain barrier, they may release their toxic cargo directly within neural tissue, creating localized high concentrations of neurotoxicants that bypass typical detoxification mechanisms (48).

### 3.4 Amyloid-beta pathology acceleration

Among the multiple mechanisms through which microplastics may contribute to neurodegeneration, their direct interaction with amyloid-beta (Aβ) protein aggregation pathways represents perhaps the most mechanistically direct link to Alzheimer’s disease pathogenesis (49). Experimental evidence demonstrates that microplastic particles, particularly nanoscale polystyrene particles, significantly influence Aβ aggregation kinetics, oligomer formation, and neurotoxicity through multiple physicochemical interactions with these pathogenic protein species (50). These findings establish a potential molecular mechanism directly connecting microplastic exposure with characteristic proteinopathic features of Alzheimer’s disease, suggesting a pathway through which environmental microplastic contamination may contribute to disease etiology or progression.

Advanced biophysical investigations utilizing thioflavin T fluorescence assays, circular dichroism spectroscopy, and atomic force microscopy have demonstrated that polystyrene nanoparticles significantly accelerate the nucleation phase of both Aβ40 and Aβ42 peptide aggregation. The nucleation process represents the rate-limiting step in amyloid formation, wherein monomeric peptides associate to form initial oligomeric seeds that subsequently template further aggregation. Quantitative kinetic analyses reveal that microplastic surfaces reduce the energy barrier for this nucleation event through multiple mechanisms. The hydrophobic surfaces of plastic particles provide favorable interfaces for peptide adsorption, effectively increasing local Aβ concentration and facilitating intermolecular interactions essential for oligomer formation. Additionally, interaction with the microplastic surface induces conformational changes in Aβ secondary structure, promoting the transition from predominantly *α*-helical and random coil configurations toward β-sheet-rich structures characteristic of amyloidogenic conformations. This surface-mediated conformational conversion accelerates the formation of initial nucleation centers from which subsequent fibril growth proceeds, effectively shortening the lag phase of amyloid formation by up to 40% in experimental systems (49).

Beyond accelerating aggregation initiation, microplastics profoundly alter Aβ aggregation pathways and resultant aggregate morphology. Comparative analyses of Aβ aggregation in the presence and absence of microplastic particles reveal that microplastic-mediated aggregation preferentially generates oligomeric and protofibrillar assemblies rather than mature amyloid fibrils (37). This altered aggregation trajectory holds significant pathophysiological implications, as considerable evidence indicates that soluble oligomeric Aβ species, rather than mature fibrils, represent the primary neurotoxic entities in Alzheimer’s disease pathogenesis. These oligomeric assemblies demonstrate enhanced capacity to disrupt neuronal membrane integrity, induce calcium dysregulation, impair synaptic function, and activate inflammatory responses compared to fibrillar species. Structural characterization of microplastic-induced Aβ oligomers reveals distinctive conformational elements and surface exposure patterns that may underlie their enhanced neurotoxicity, including increased exposure of hydrophobic residues that facilitate membrane interaction and altered epitope accessibility that modulates recognition by clearance mechanisms (49, 51).

Microplastic-Aβ interactions additionally influence the stability and persistence of neurotoxic assemblies. Experimental evidence indicates that microplastic surfaces can stabilize toxic conformations of Aβ oligomers through continuous surface interactions that prevent their conversion to less toxic fibrillar forms or dissociation back to monomeric species. This stabilization effectively prolongs the biological half-life of oligomeric assemblies, extending their window of neurotoxic activity within the neural parenchyma. Surface analytical techniques including quartz crystal microbalance with dissipation monitoring and surface plasmon resonance demonstrate that various microplastic polymer compositions exhibit differential binding affinities for Aβ species, suggesting polymer-specific effects on oligomer stabilization that may contribute to variable neurotoxic potential among different microplastic types. Additionally, the microplastic surface may protect bound Aβ assemblies from proteolytic degradation by physically restricting access of clearance enzymes including neprilysin and insulin-degrading enzyme to their peptide substrates, further enhancing oligomer persistence.

**Figure 2:**
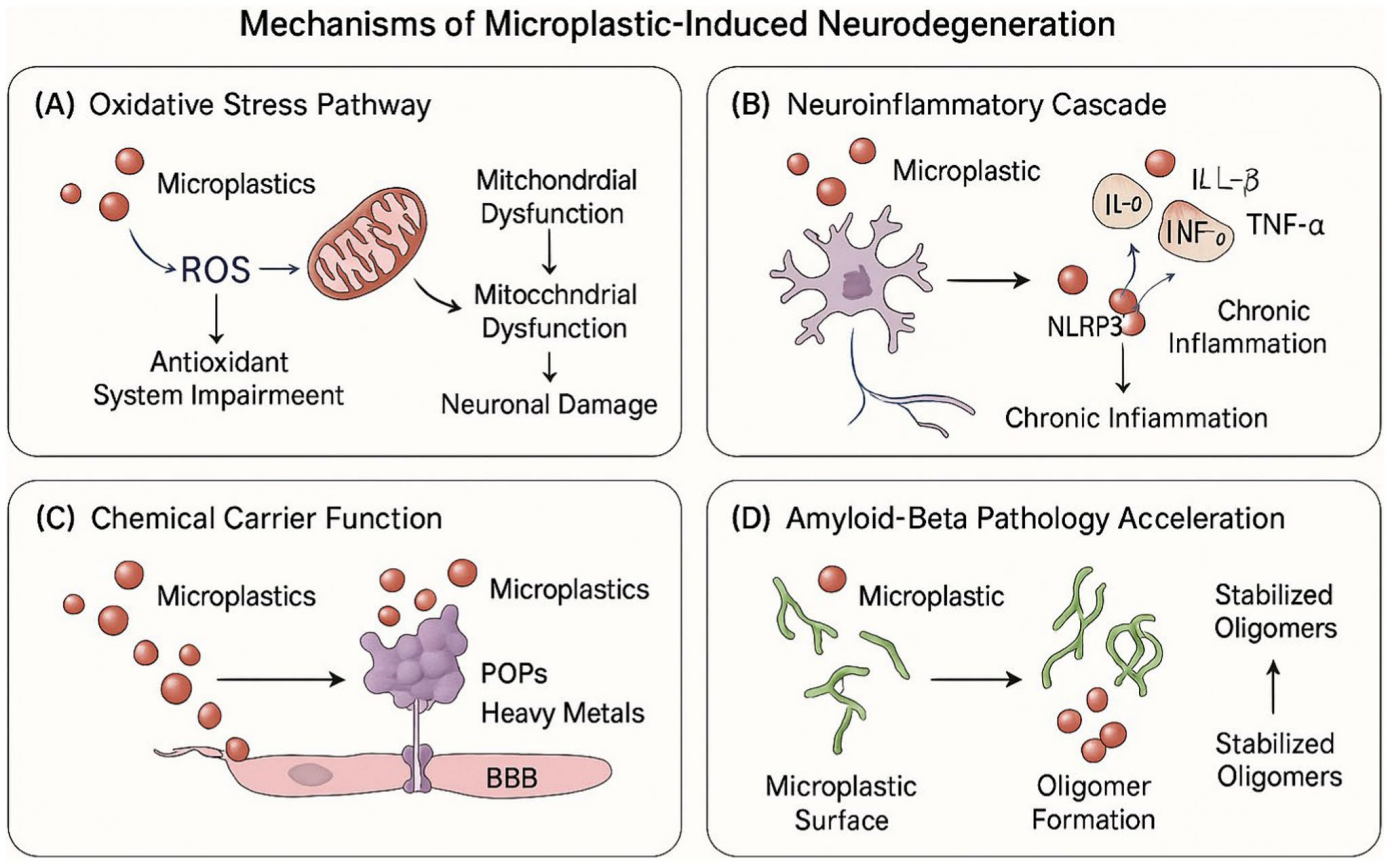
Mechanisms of microplastic-induced neurodegeneration. The figure depicts the four primary pathways through which microplastics contribute to neurodegeneration: **(A)** oxidative stress pathway – showing microplastic-induced ROS production, mitochondrial dysfunction, and antioxidant system impairment leading to neuronal damage; **(B)** neuroinflammatory Cascade – illustrating microglial activation, pro-inflammatory cytokine release, NLRP3 inflammasome activation, and chronic inflammation; **(C)** chemical carrier function – demonstrating how microplastics transport POPs, heavy metals, and plastic additives across the BBB; and **(D)** amyloid-beta pathology acceleration – showing microplastic surface interactions with Aβ peptides facilitating oligomer formation and stabilization.

These microplastic-amyloid interactions hold particular significance given the central role of Aβ oligomerization in Alzheimer’s disease pathogenesis and the demonstrated capacity of microplastics to penetrate the blood–brain barrier and accumulate within neural tissue. The co-localization of microplastic particles with regions of Aβ deposition in post-mortem analyses of dementia-affected brain tissue provides circumstantial evidence supporting the pathophysiological relevance of these interactions. Furthermore, the preferential accumulation of microplastics in perivascular spaces coincides with regions of enhanced Aβ clearance through the glymphatic system, suggesting potential interference with physiological amyloid clearance mechanisms that may compound direct effects on aggregation kinetics. Collectively, these findings establish a mechanistic framework whereby microplastic exposure may directly modulate a central pathogenic process in Alzheimer’s disease, providing a molecular link between environmental exposure and neurodegenerative progression (52). As illustrated in Figure 2, these four interconnected pathogenic mechanisms collectively contribute to microplastic-induced neurodegeneration through complementary molecular and cellular pathways, potentially accelerating dementia pathogenesis in exposed individuals.

## 4 Bioaccumulation of microplastics in human brain tissue

Recent research by Nihart et al. (53) documented significant microplastic accumulation in human brain tissue through a comprehensive analytical protocol employing Py-GC/MS, ATR-FTIR spectroscopy, and electron microscopy with energy-dispersive spectroscopy. Quantitative assessment demonstrated pronounced differential distribution across tissues, with frontal cortex specimens containing 3,345–4,917 μg/g (median concentration)—substantially exceeding concentrations in liver or kidney tissue by factors of 7–30. Polymer composition analysis revealed predominance of polyethylene (75% of total microplastic content) with additional polymers (polypropylene, polyvinyl chloride, styrene-butadiene rubber) present at lower concentrations. Notably, specimens from subjects with confirmed dementia diagnoses exhibited significantly elevated microplastic burden (26,076 μg/g median concentration), representing a five-fold increase compared to age-matched non-dementia controls—an association that remained statistically significant after multivariate adjustment for potential demographic and clinical confounders (53).

Research by Campen et al. (54) provides quantitative documentation of microplastic accumulation in human neural tissue through comprehensive analytical methodology. Using pyrolysis gas chromatography–mass spectrometry (Py-GC/MS), investigators identified significant tissue-specific microplastic distribution, with frontal cortex specimens demonstrating concentrations (3,345–4,917 μg/g) that substantially exceeded hepatic and renal tissues by 7–30 fold. Polymer compositional analysis revealed polyethylene predominance (74%) within neural tissue (54).

## 5 Research challenges and future directions

Despite emerging evidence linking microplastic exposure to potential neurodegeneration, significant methodological and knowledge limitations constrain our comprehensive understanding of this relationship. These constraints necessitate strategic research initiatives to establish causal mechanisms and inform preventive interventions.

### 5.1 Methodological limitations

Analytical methods for microplastic detection and quantification in biological matrices remain in developmental stages, with inconsistent standardization across research protocols. This methodological heterogeneity generates variable results between studies, significantly complicating cross-study comparisons and meta-analytical integration (38). The detection of nanoplastic particles (<1 μm) represents a particular analytical challenge, as conventional techniques demonstrate diminishing sensitivity with decreasing particle dimensions. This limitation is especially consequential given that nanoplastics may possess enhanced neurological significance due to their superior blood–brain barrier penetration capacity. Furthermore, the ubiquitous environmental prevalence of microplastics introduces substantial contamination risks throughout analytical workflows. Sample collection, processing, and analysis protocols require rigorous quality control measures including clean room environments, procedural blanks, and specialized equipment to ensure data validity, adding substantial methodological complexity to research initiatives.

### 5.2 Knowledge gaps

Comprehensive human exposure assessment across diverse populations constitutes a critical knowledge deficit. Current understanding of microplastic exposure lacks adequate characterization across geographical regions, socioeconomic strata, and occupational settings, limiting the development of population-level risk profiles and targeted intervention strategies. The heterogeneous nature of microplastic composition introduces additional complexity, as different polymer types may exhibit distinct toxicological profiles, blood–brain barrier penetration efficiencies, and biological persistence characteristics. These polymer-specific differences remain inadequately characterized despite their fundamental importance for accurate risk assessment. Physical properties including particle size, morphology, and surface characteristics significantly influence microplastic toxicity through modulation of cellular internalization mechanisms, immune recognition pathways, and neuroinflammatory potential, yet these dimensional structure–activity relationships remain poorly defined. Environmental and biological exposures invariably occur as complex microplastic mixtures with diverse polymer compositions, varied size distributions, and heterogeneous associated chemicals, creating potential for additive, synergistic, or antagonistic effects that current single-polymer experimental models inadequately capture. Longitudinal datasets tracking microplastic exposure in relation to cognitive outcomes remain notably absent from the literature, yet such temporal data are essential for establishing causality and elucidating latency periods between exposure and neurodegeneration.

### 5.3 Future research priorities

Addressing these limitations requires strategic research initiatives encompassing methodological standardization, epidemiological investigation, and mechanistic elucidation. Development and validation of standardized analytical protocols for microplastic detection in biological samples represent an immediate priority, with particular emphasis on enhancing nanoplastic detection sensitivity through advanced spectroscopic and chromatographic approaches. Longitudinal epidemiological studies incorporating repeated microplastic exposure assessments with comprehensive cognitive evaluation would establish temporal precedence and dose–response relationships critical for causal inference. Detailed mechanistic investigations utilizing advanced *in vitro* and *in vivo* models should characterize molecular and cellular pathways mediating microplastic neurotoxicity, with particular focus on brain region-specific vulnerabilities and cell type-specific responses to various polymer compositions. Systematic comparative evaluation of different polymer types, sizes, and morphologies would identify materials posing greatest neurological risk, informing regulatory prioritization and material substitution initiatives. Development and validation of exposure mitigation strategies spanning environmental remediation, industrial emission controls, and personal protective approaches would translate research findings into practical interventions reducing neurological risk. Identification of validated biomarkers for microplastic exposure and early neurological effects would facilitate exposure monitoring, high-risk population identification, and early intervention implementation. Integration of microplastic analysis within established air pollution and dementia research frameworks would enhance methodological efficiency while potentially identifying synergistic effects between environmental exposures that current single-contaminant models fail to capture.

## 6 Conclusion

The emerging evidence linking microplastic exposure to potential neurodegeneration represents a significant development in our understanding of environmental risk factors for dementia. Through multiple pathogenic mechanisms including oxidative stress induction, neuroinflammation promotion, neurotoxic chemical transport, and amyloid-beta pathology acceleration, microplastics may contribute to the complex etiology of dementia. Recent findings confirming microplastic bioaccumulation in human brain tissue, with notably higher concentrations in individuals with dementia, lend support to this hypothesis.

However, considerable research challenges and knowledge gaps remain. Standardized methodologies, comprehensive exposure assessment, detailed mechanistic understanding, and longitudinal epidemiological data are needed to establish microplastics as a causative factor in dementia pathogenesis and to inform effective intervention strategies.

Given the rapidly increasing environmental microplastic burden and the projected rise in dementia prevalence, addressing these research priorities has significant implications for both environmental policy and public health (51). If confirmed through rigorous investigation, microplastic exposure may represent a novel modifiable risk factor for dementia, offering new opportunities for prevention strategies and highlighting the interconnectedness of environmental and neurological health.
